# Supplementary material for: Protein-directed ribosomal frameshifting temporally regulates gene expression
Source: Nat Commun. 2017 Jun 8;8:15582. doi: 10.1038/ncomms15582 (PMC5472766; doi:10.1038/ncomms15582)
Supplement: Supplementary Information — Supplementary Table and Supplementary Figures [file ncomms15582-s1.pdf]

SUPPLEMENTARY INFORMATION

Supplementary Table 1. Host and virus read counts for different samples.

virus	time point	protocol	total reads	host rRNA	host mRNA	vRNA (+)
**Round 1**						
WT	2 h p.i.	Ribo-Seq	41,600,458	39,428,673	935,243	1,273
WT	4 h p.i.	Ribo-Seq	43,631,852	40,177,463	1,375,172	46,645
WT	6 h p.i.	Ribo-Seq	40,726,538	37,302,246	756,724	527,689
WT	8 h p.i.	Ribo-Seq	39,825,558	37,307,288	193,188	384,185
SS	2 h p.i.	Ribo-Seq	50,369,240	48,072,231	1,041,989	1,652
SS	4 h p.i.	Ribo-Seq	47,535,292	43,669,060	1,412,049	44,563
SS	6 h p.i.	Ribo-Seq	49,746,578	47,480,164	496,718	215,017
SS	8 h p.i.	Ribo-Seq	48,745,605	46,892,840	175,197	226,584
**Round 2**						
WT	8 h p.i.	Ribo-Seq	24,226,113	20,893,697	130,814	310,264
SS	8 h p.i.	Ribo-Seq	23,077,732	20,543,723	65,341	201,538
WT-SL	8 h p.i.	Ribo-Seq	29,021,722	25,730,834	37,179	294,600
SS-SL	8 h p.i.	Ribo-Seq	30,213,321	27,011,742	72,438	314,263
LV-WT	8 h p.i.	Ribo-Seq	19,827,674	17,112,687	47,257	184,811
LV-SS-SL	8 h p.i.	Ribo-Seq	37,213,678	32,526,139	66,372	391,593
WT	8 h p.i.	RNA-Seq	26,972,824	193,453	1,273,930	8,592,537
SS	8 h p.i.	RNA-Seq	31,164,415	268,151	1,128,866	10,654,802
WT-SL	8 h p.i.	RNA-Seq	31,808,988	171,530	796,828	11,578,854
SS-SL	8 h p.i.	RNA-Seq	31,301,691	264,905	921,203	12,216,753
LV-WT	8 h p.i.	RNA-Seq	28,484,935	210,335	1,223,020	5,928,352
LV-SS-SL	8 h p.i.	RNA-Seq	33,754,451	304,986	1,051,899	9,706,187

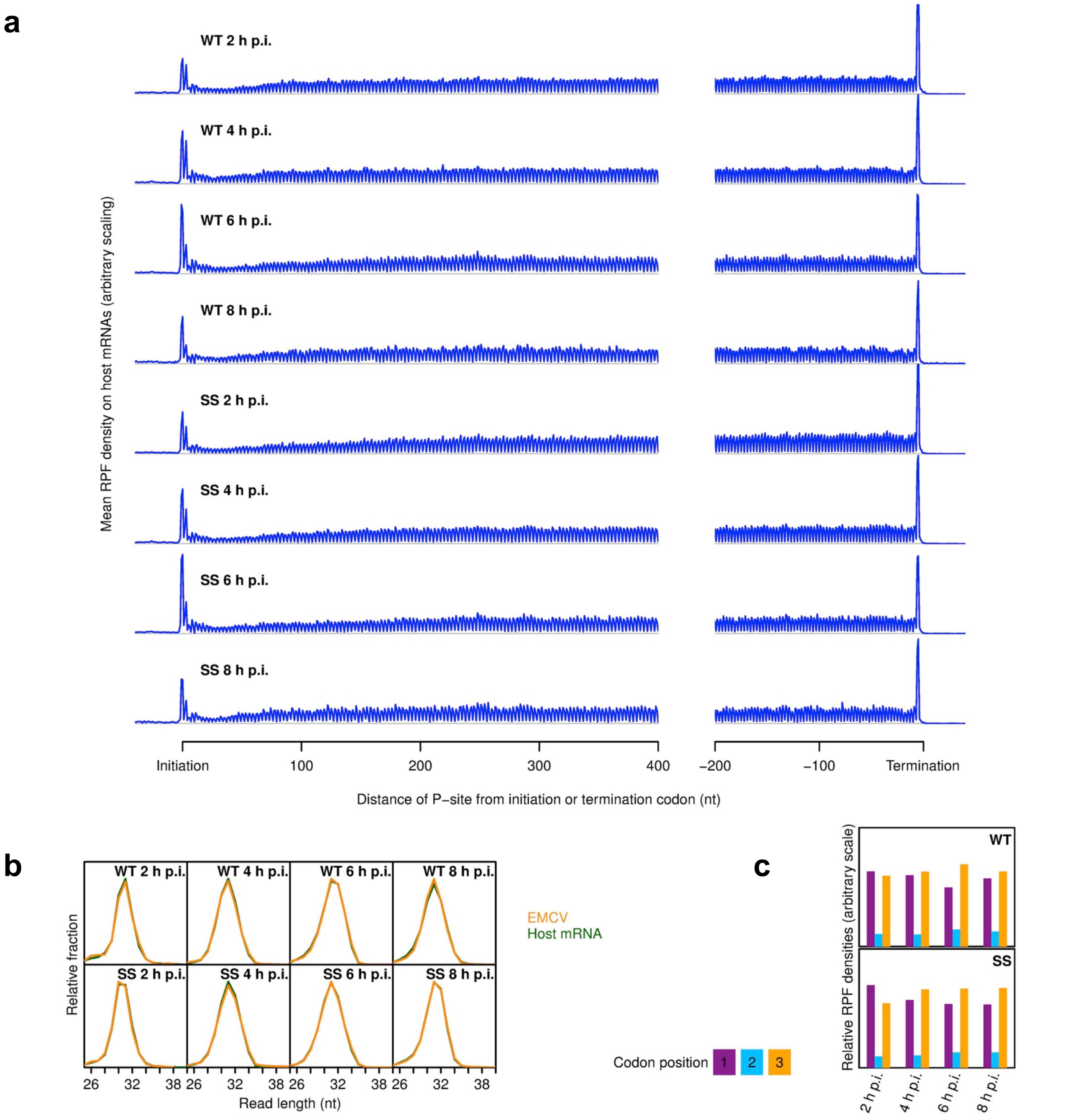

Supplementary Figure 1. Assessment of ribosome profiling quality. (**a**) Histograms of RPF inferred P-site positions relative to annotated initiation and termination sites summed over all host mRNAs. (**b**) Relative length distributions for Ribo-Seq reads mapping to virus (orange) and host (green) mRNAs. (**c**) Phasing of 5′ ends of Ribo-Seq reads that map to host mRNA coding regions.

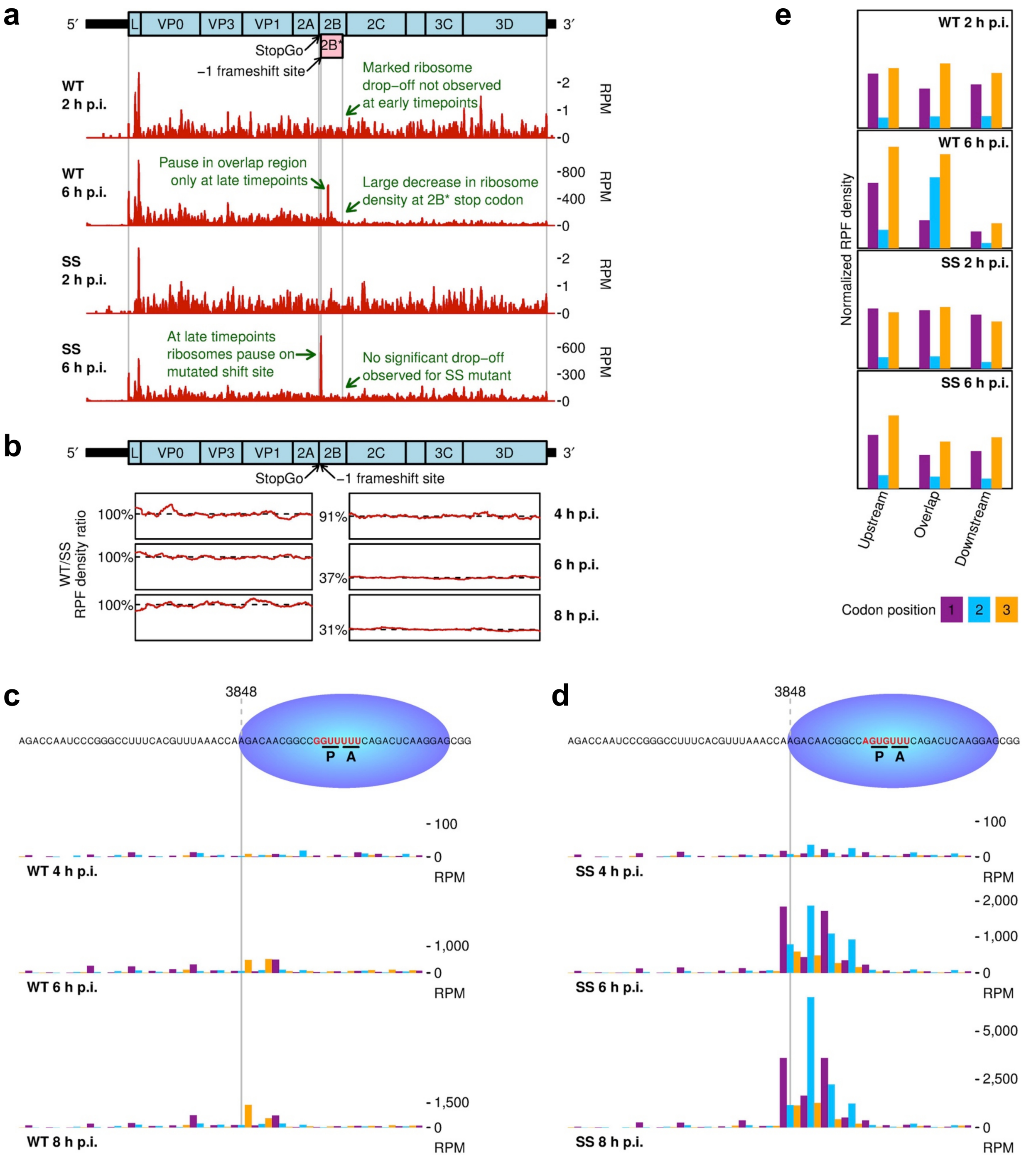

Supplementary Figure 2. Ribosomes pause on a mutated frameshift site. (**a**) Ribo-Seq RPF densities in reads per million mapped reads (RPM) on WT and SS virus genomes at 2 and 6 h p.i. (see Fig. 1d for 4 and 8 h p.i.). (**b**) Ratio of WT and SS RPF densities after first smoothing with a 225-nt running-mean filter (normalization by SS factors out nucleotide-to-nucleotide variations in translational kinetics and technical biases). UTRs were excluded and also the 2B/2B* overlap region (since, in this region, the WT and SS RPFs distributions are quite different due to ribosomes translating predominantly different reading frames in the two viruses). Percentage values are based on division of the mean levels in each region (downstream/upstream). One minus these values estimates the PRF efficiency (i.e. 9, 63 and 69% at 4, 6 and 8 h p.i. repectively). (**c-d**), Extent of ribosome pausing at the frameshift site in WT virus (c) and SS mutant virus (d). The grey line indicates the position of the RPF 5′ end when the ribosome (schematically illustrated by a blue ellipse) is positioned with P- and A-site codons as indicated. Red letters indicate the WT (c) or mutated (d) shift site. Note that nucleotide-to-nucleotide variation in RPF counts may be influenced

by RPF phasing bias and technical biases. The height of each histogram on the page is scaled by the sum of RPFs mapping anywhere within the viral genome, while the y-axis shows counts per million mapped reads (RPM). (**e**) Phasing of RPFs mapping upstream of 2B*, within the 2B/2B* overlap region, and downstream of 2B*.

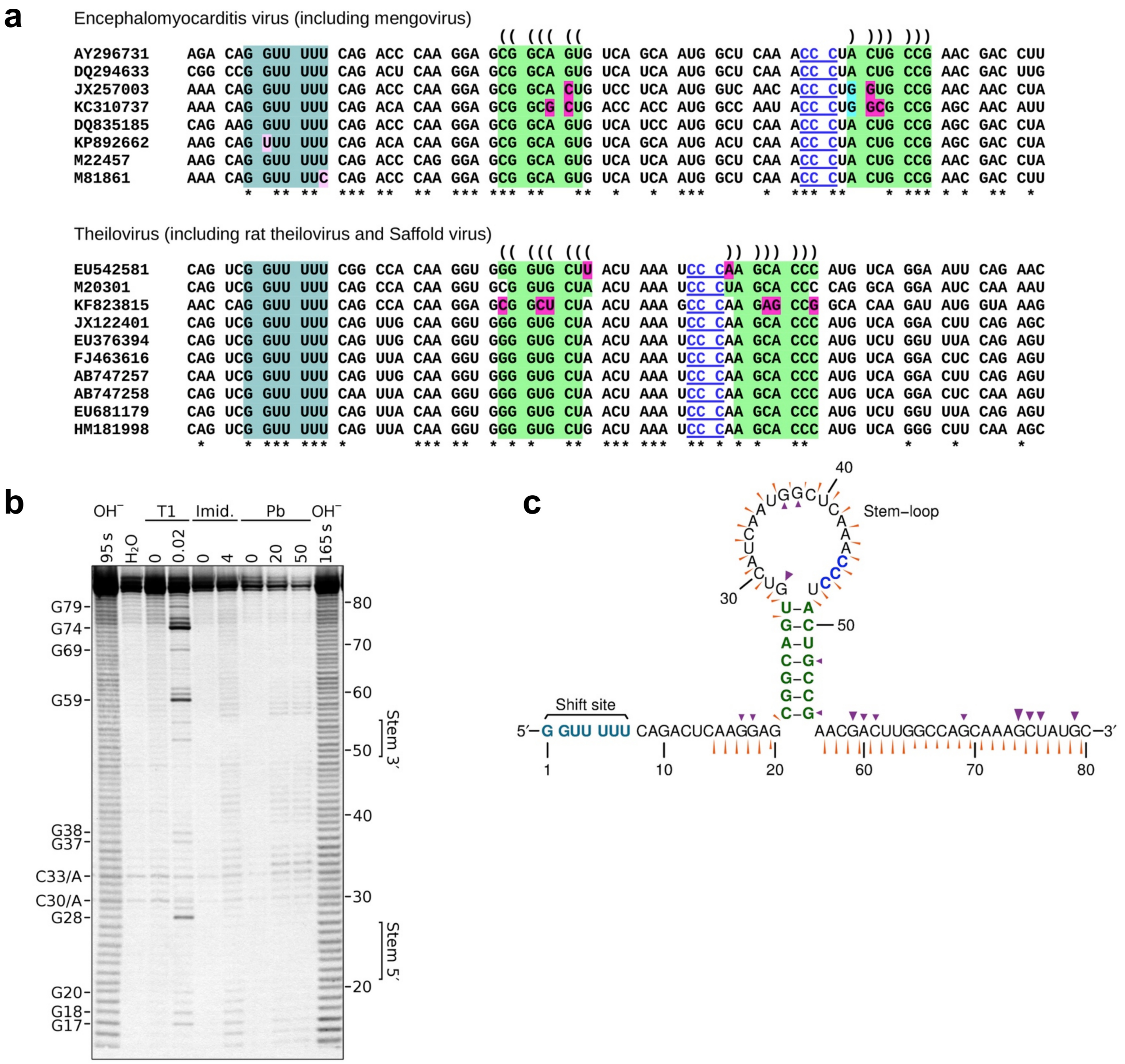

Supplementary Figure 3. A 3′ stem-loop structure is conserved in cardioviruses. (**a**) Sequence of the PRF region from representative cardiovirus isolates, showing the highly conserved shift site (slate blue) and 3′ RNA stem-loop structure (green). Nucleotide variations in the shift site are indicated in pale pink and are compatible with other −1 PRF shift site sequences[1]. Parentheses indicate predicted base-pairings. Substitutions that preserve the predicted base-pairings are highlighted in pink. The conserved CCC loop triplet is underlined. (**b**) RNA was 5′-end-labelled with [γ-^{33}P]ATP and subjected to limited RNase or chemical cleavage using structure-specific probes. Sites of cleavage were identified by comparison with a ladder of bands created by limited alkaline hydrolysis of the RNA (OH$^-$; RNA heated to 100 °C for 95 or 165 s as indicated), and the positions of cuts by RNase T1 which preferentially cleaves at G bases in single-stranded regions (T1; units of enzyme added). Uniquely cleaved nucleotides were identified by their absence in untreated control lanes (0). Chemical structure probing was with imidazole (Imid.; numbers indicate hours), or lead acetate (Pb; mM concentration in reaction), which show specificity for single-stranded regions. The H_2O lane represents RNA dissolved in water, incubated for 4 h, and processed in parallel to the imidazole-treated sample. Cleavage by lead and imidazole indicated that, under the experimental conditions used, much of the RNA is in a single-stranded conformation. However, there was evidence for base-pairing in two regions indicated by stem 5′ and stem 3′. (**c**) The reactivities of RNase T1 (purple arrowheads) and imidazole (thin orange arrowheads) are indicated on a schematic of the EMCV PRF signal showing the evolutionarily conserved stem-loop structure. The lead acetate

cleavages are similar to imidazole. The size of the symbols is approximately proportional to the intensity of cleavage at that site.

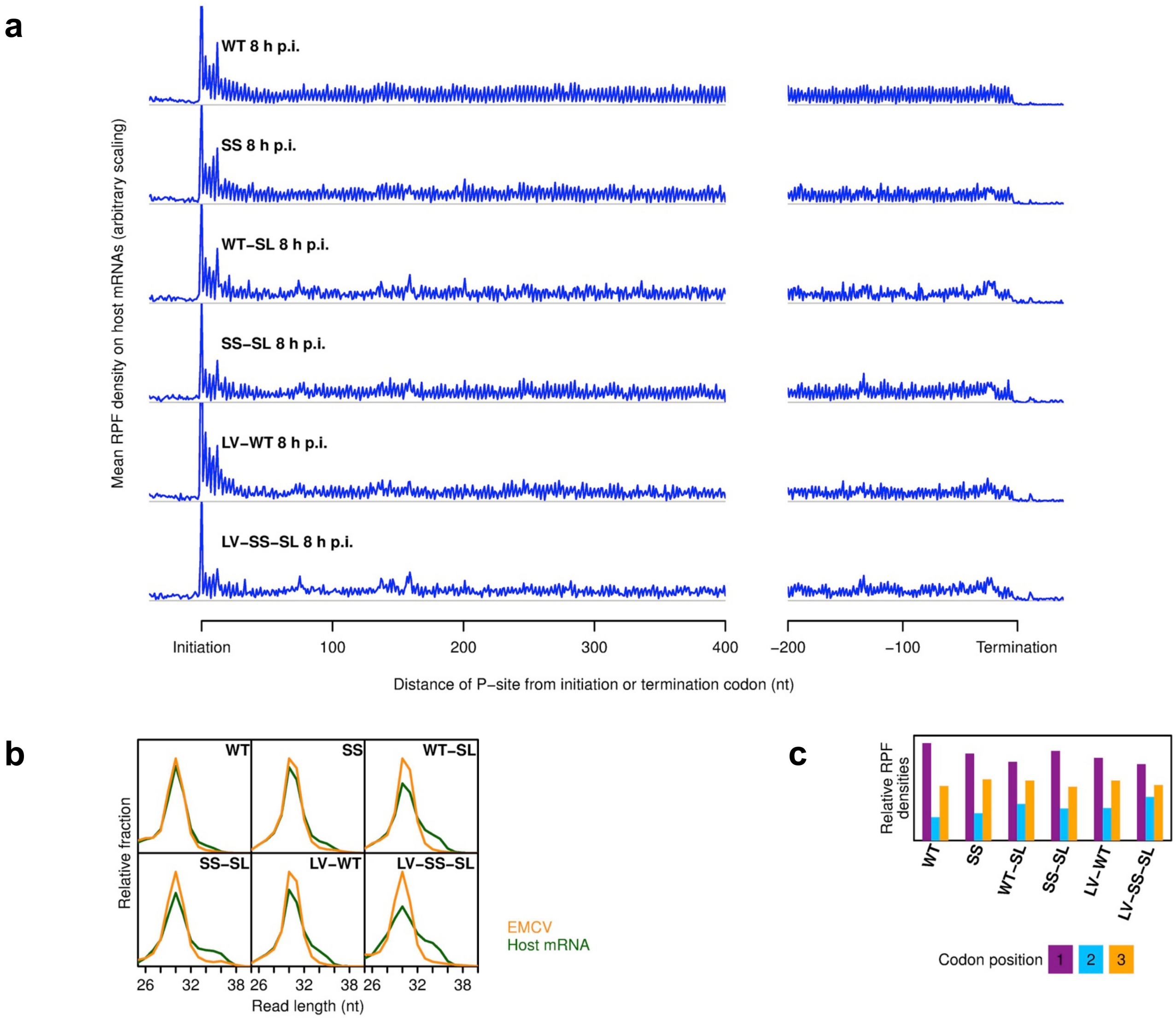

Supplementary Figure 4. Assessment of ribosome profiling quality. (**a**) Histograms of RPF inferred P-site positions relative to annotated initiation and termination sites summed over all host mRNAs. (**b**) Relative length distributions for Ribo-Seq reads mapping to virus (orange) and host (green) mRNAs. Note that while the virus Ribo-Seq read length distribution is sharply peaked at ~30 nt, the host distribution has a high-end shoulder that may be indicative of some level of non-RPF contamination, potentially due to the cytopathic effects of virus infection. (**c**) Phasing of 5′ ends of Ribo-Seq reads that map to host mRNA coding regions.

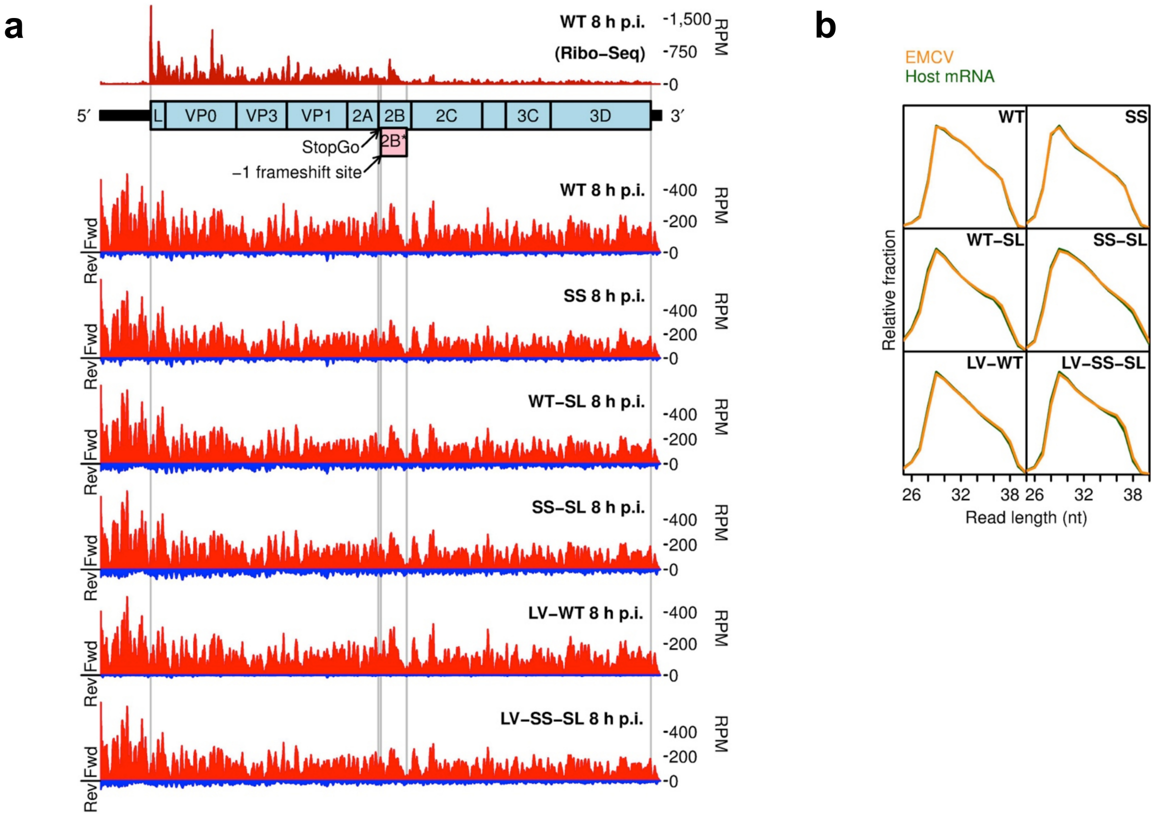

Supplementary Figure 5. RNA-Seq analysis of WT and mutant viruses. (**a**) RNA-Seq read densities in reads per million mapped reads (RPM) on WT and mutant virus genomes at 8 h p.i. Reads mapping to positive- and negative-sense viral RNA are shown in red and blue respectively. The 8 h p.i. WT virus Ribo-Seq sample from Fig. 2b is also shown at the top for comparison. Note that a local dip in RPF density around the 2B* stop codon masks the true site of ribosome drop-off but is present also in RNA-Seq and therefore presumably derives from sequencing biases. (**b**) Relative length distributions for RNA-Seq reads mapping to virus (orange) and host (green) mRNAs.

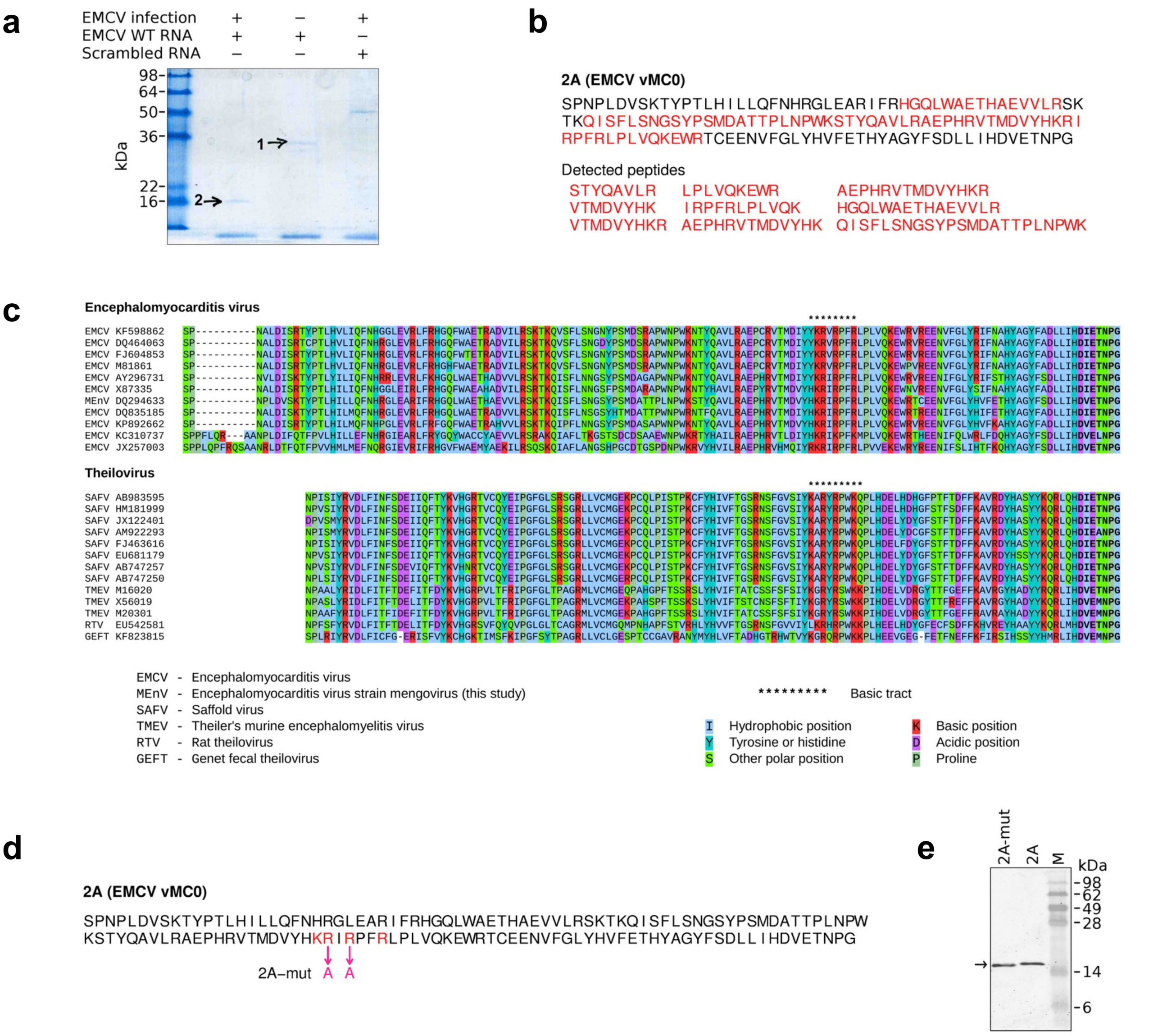

Supplementary Figure 6. Viral protein 2A binds to the stem-loop RNA. (**a**) 5-bromo-uridine labelled RNA containing the WT or a scrambled version of the EMCV PRF signal was incubated with lysate from EMCV-infected or mock-infected cells, as indicated. Bound proteins were eluted after washing, resolved by SDS-PAGE, and stained with colloidal Coomassie blue. The indicated bands were excised from the gel, subjected to in-gel trypsin digest, and analyzed by liquid chromatography tandem mass spectrometry (LC-MS/MS). Band 1 was identified as alpha-S1/S2-casein precursor, a common lab contaminant (cRAP database CAS1_BOVIN/CAS2_BOVIN; www.thegpm.org). Band 2 was identified as the EMCV 2A protein. (**b**) Complete amino acid sequence of the EMCV 2A protein. Tryptic peptides identified by mass spectrometry of the gel slice containing band 2 are indicated in red. No other EMCV peptides were detected in the gel slice. (**c**) Alignment of 2A amino acid sequences from representative cardiovirus isolates. (**d**) Arg to Ala mutations introduced into EMCV 2A at residues 95 and 97. (**e**) Anti-2A western blot showing expression of recombinant 2A and 2A-mut proteins; note that 2A-mut migrates slightly faster than 2A.

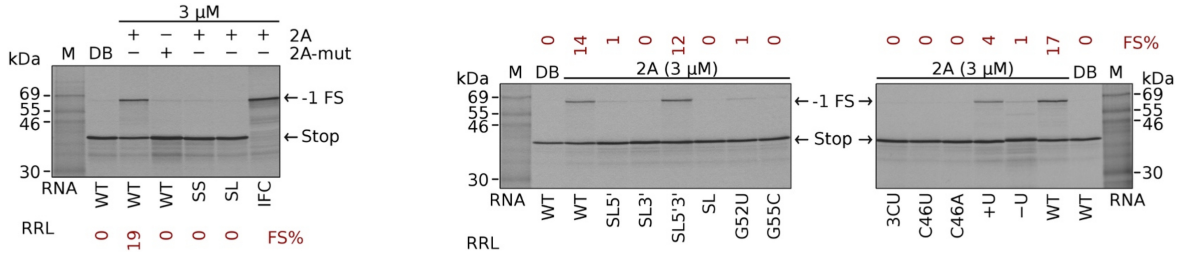

Supplementary Figure 7. Analysis of the EMCV frameshift signal. RNAs derived from *Fsp*I-cut plasmid were translated in rabbit reticulocyte lysate (RRL) in the presence of 3 μM 2A or 2A-mut, or 2A dialysis buffer (DB). Products generated by ribosomes that do not frameshift (stop) or that enter the −1 reading frame (−1 FS) are indicated. M and IFC indicate markers and the in-frame control, respectively.

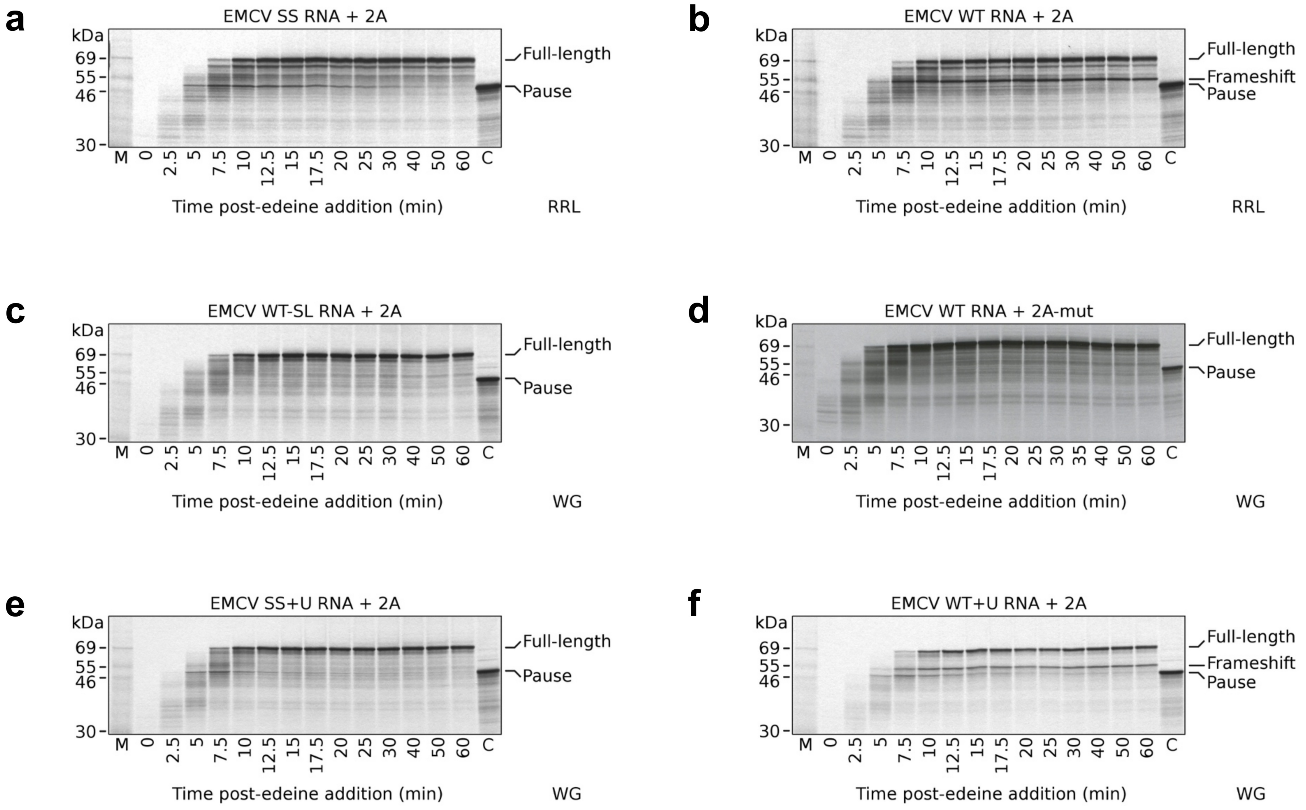

Supplementary Figure 8. Ribosomal pausing at the EMCV frameshift signal. (**a-f**) RNAs derived from *Ava*II-cut plasmids were translated in RRL (a-b) or WG extract (c-f) for 5 min prior to addition of edeine. Aliquots were removed at various times post-edeine addition, and products resolved by SDS-PAGE. Lanes M and C show markers and the expected size of the ribosomal pause product, respectively. Translations were supplemented with 1 μM 2A (a-c, e-f) or 2A-mut (d). As well as the full-length product and the transient pausing product, a frameshift product is produced for WT and WT+U RNA (b and f).

REFERENCES

1. Atkins, J. F., Loughran, G., Bhatt, P. R., Firth, A. E. & Baranov, P. V. Ribosomal frameshifting and transcriptional slippage: from genetic steganography & cryptography to adventitious use. *Nucleic Acids Res.* **44,** 7007-7078 (2016).
